# What is the relationship between type 2 diabetes mellitus status and the neuroradiological correlates of cerebral small vessel disease in adults? Protocol for a systematic review

**DOI:** 10.1186/s13643-017-0410-1

**Published:** 2017-01-17

**Authors:** Clark Funnell, Mary M. Doyle-Waters, Samuel Yip, Thalia Field

**Affiliations:** 1 Department of Medicine, Division of Neurology, University of British Columbia, S169-2211 Wesbrook Mall, Vancouver, BC V6T 2B5 Canada; 2 Centre for Clinical Epidemiology and Evaluation, Research Pavilion, 708A-828 West 10th Avenue, Vancouver, BC V5Z 1M9 Canada; 3 Department of Medicine, Division of Neurology, University of British Columbia, 8278-2775 Laurel St., Vancouver, BC V5Z 1M9 Canada

**Keywords:** Systematic review, Cerebral small vessel disease, Stroke, Magnetic resonance imaging, Type 2 diabetes mellitus, White matter hyperintensities, Lacunes, Cerebral microbleeds, Perivascular spaces

## Abstract

**Background:**

Cerebral small vessel disease (CSVD) is a common cause of stroke, dementia, and functional decline. In recent years, neuroradiologic correlates of CSVD have been identified. These imaging findings, best characterized on magnetic resonance imaging (MRI), include some combination of white matter hyperintensities, lacunes, cerebral microbleeds, enlarged perivascular spaces, and cerebral atrophy. Though some cohorts have reported that participants with type 2 diabetes mellitus (T2DM), an important risk factor for CSVD, may have a distinct neuroradiologic phenotype, this relationship is not well-characterized. Adults with diabetes mellitus have a two- to threefold higher incidence of ischemic stroke compared to controls and are an increasingly important population given global trends of increasing diabetes prevalence. This study aims to determine if adults with CSVD and T2DM have a distinct neuroradiologic phenotype.

**Methods:**

A systematic search of the literature will be conducted to find articles that report the MRI features of CSVD in a cohort of participants including those with and without type 2 diabetes mellitus (T2DM). A number of databases will be searched including MEDLINE, Embase, CINAHL, and Web of Science. Proceedings and abstracts from key conferences will also be reviewed and relevant journals hand searched for additional papers. The references from selected papers will be scanned. Screening of potential articles, data extraction, and quality appraisal will be performed in duplicate by independent reviewers. Odds ratios and 95% confidence intervals for the presence versus absence of each neuroradiologic correlate of interest from each included study will be calculated. If sufficient homogeneity exists among studies, a meta-analysis will be performed for each neuroradiologic correlate of CSVD. If heterogeneity of studies precludes data pooling, results will be presented in narrative form.

**Discussion:**

Determining whether a distinct neuroradiologic phenotype of CSVD exists in adults with T2DM will provide insight into the underlying mechanisms of CSVD and guide future research on therapeutic targets.

**Systematic review registration:**

PROSPERO CRD42016046669

**Electronic supplementary material:**

The online version of this article (doi:10.1186/s13643-017-0410-1) contains supplementary material, which is available to authorized users.

## Background

Cerebral small vessel disease (CSVD) is a neurodegenerative condition affecting the small blood vessels of the brain. In contrast to cerebral large vessels, small vessels are not visualized by contemporary imaging methods and therefore cerebral small vessel disease is used to describe the parenchyma lesions rather than the underlying small vessel alterations [1]. The neuroimaging correlates of CSVD include lacunar infarcts, white matter hyperintensities, enlarged perivascular spaces, microbleeds, and brain atrophy [2]. CSVD is a neuroradiological diagnosis and the above findings may occur in adults with or without a history of clinically manifest stroke or dementia [3]. However, the presence and severity of CSVD neuroimaging correlates are associated with risk factor burden, baseline cognition and function, and prognosis with respect to recurrent stroke and cognitive decline [4–6].

CSVD is estimated to affect 250,000 people in Canada alone and this number is anticipated to rise as the population ages. CSVD contributes to 20% of ischemic stroke [3, 4] and 45% of dementias and the current estimated direct (medical expenses) and indirect (lost productivity) costs of stroke and dementia in Canada exceeds $30 billion annually [6, 7]. There are few known effective therapies for CSVD. Treatment remains empiric and is directed at controlling vascular risk factors including hypertension, dyslipidemia, smoking, and hyperglycemia. Unlike large artery disease, these conventional risk factors may explain only a small proportion of CSVD, highlighting the need for targeted therapies.

It is not known whether there is a sequential progression of neuroradiological findings of CSVD, where the appearance of certain imaging features consistently precedes others [7]. Further, certain neuroimaging features of CSVD seem to co-aggregate more consistently than others [5, 8]. Insights regarding risk factors for these separate “phenotypes” of CSVD may yield novel insights with respect to the mechanism of this disease and direct therapeutic strategies in the future.

People with diabetes are an important population given the increasing global burden of the disease (estimated at over 400 million) and the substantially elevated incidence of stroke (two- to threefold higher) in people with diabetes compared to controls without diabetes [9]. In the recent Secondary Prevention of Small Subcortical Strokes (SPS3) trial, which included participants with recent lacunar infarcts, those with a history of diabetes had distinct neuroimaging characteristics on magnetic resonance imaging (MRI) as compared with those without diabetes, with an increased odds of posterior circulation infarcts and a lower burden of microbleeds and enlarged perivascular spaces [10–12]. Whether this phenotype is consistently observed in other cohorts is not known.

The most recent systematic review examining brain imaging findings in adults with diabetes excludes discussion of findings such as microbleeds and enlarged perivascular spaces that have only more recently been widely described in larger contemporary cohorts in the medical literature [13]. Furthermore, this review combined findings from both MRI and computed tomography (CT) studies, which introduces substantial heterogeneity as CT has reduced sensitivity for detecting findings of CSVD, in particular mild-to-moderate white matter hyperintensities, cerebral microbleeds, or enlarged perivascular spaces. Given that a distinct neuroimaging phenotype of CSVD may underlie distinct pathophysiological mechanisms and therapeutic strategies in adults with T2DM, we will perform an updated systematic review of the literature to compare MRI neuroimaging features of CSVD in adults with versus T2DM.

### Aim

To determine whether a unique neuroradiologic phenotype of CSVD is found in adults with T2DM. This will be achieved by comparing the presence and severity versus absence of features of cerebral small vessel disease (white matter hyperintensities, lacunar infarcts, cerebral microbleeds, and/or enlarged perivascular spaces) on MRI in adults with T2DM versus adults without T2DM. This protocol conforms to the PRISMA-P guidelines [14] [see Additional file 1].

### Inclusion and exclusion criteria

Our research question does not strictly align with the traditional “Patient, Intervention, Comparison, Outcome” (PICO) format. For clarity, we will separately describe the inclusion and exclusion criteria for study participants, MRI scans, exposure (diabetes mellitus, as described below), and outcomes separately.

### Participants

#### Participants

Studies of interest are those which include adults aged 18 or older receiving brain MRI for any reason. Studies must include a group of patients with and without diabetes mellitus. Patients in either group with other risk factors for CSVD (i.e., hypertension, dyslipidemia, cigarette smoking) shall not be excluded.

#### MRI

The MRI scanner type and the sequences obtained must be stated in the paper or data supplement. If such information is not described, we will attempt to contact the authors to obtain this information. If the information cannot be obtained, the study would be excluded. The sequences obtained must be deemed by the reviewers to be acceptable for determination of the imaging features reported in the study (see Table 1).

**Table 1:** Description of neuroimaging features of cerebral small vessel disease on MRI

	International consensus definition [2]	Common rating scales/definitions	Examples of features counted as “present” in review
White matter hyperintensities (WMH)	Signal abnormality of variable size in the white matter with hyperintensity on T2-weighted images without cavitation	• Age-related white matter changes (ARWMC) score • Fazekas score • Scheltens score • Quantitative volumetric measurements	• ARWMC score of 5 or greater (moderate–severe) • Fazekas periventricular = 3 and/or deep ≥2 • WMH volume ≥7.7 mL [3]
Lacunar infarcts	Round/ovoid subcortical fluid-filled cavity 3–15 mm in diameter in the territory of one perforating arteriole		≥1 lacune
Cerebral microbleeds (CMB)	Small (≤10 mm) areas of signal void with associated blooming on T2*-MRI or other sequences sensitive to susceptibility effects	• Brain observer microbleed score (BOMBS) • Microbleed anatomical rating scale (MARS)	≥1 microbleed
Enlarged perivascular spaces (PVS)	Fluid-filled spaces following the typical course of a vessel through grey or white matter, isointense to CSF on all sequences. Linear when images parallel to the vessel and round or ovoid when perpendicular. In constrast with lacunes, no T2-hyperintense rim on T2/FLAIR unless they traverse WMH	• Edinburgh score	

Acceptable MRI modalities for assessing CSVD features include T1 and T2/T2 fluid-attenuated inversion recovery (FLAIR) imaging for white matter hyperintensities, lacunes, and enlarged perivascular spaces and either T2*-gradient echo or susceptibility-weighted imaging (SWI) for cerebral microbleeds [1].

### Exposure

The exposure being considered is presence of diabetes. The method used to identify patients with diabetes must be stated in the paper. To maximize sensitivity, multiple definitions of diabetes status will be accepted; those based on fasting blood glucose, oral glucose tolerance test, hemoglobin A1c, use of anti-diabetes medications, or physician/hospital records will be deemed acceptable. Studies which do not include a description of how patients with diabetes were identified will be excluded. Specification of the duration of diabetes or a measure of disease severity (hemoglobin A1c, presence of end-organ damage, or history of ketoacidosis or hyperosmolar state [15]) will not be required for inclusion, though this information will be extracted if available and will contribute towards assessment of risk of bias. If specifically identified, participants with type 1 diabetes shall be excluded and if diabetes type is not specified in the paper, the authors will be contacted for clarification.

### Outcomes

Primary outcomes include the presence of MRI features of CSVD (any of: white matter hyperintensities, lacunar infarcts, cerebral microbleeds and/or enlarged perivascular spaces). Secondary outcomes focus on the severity of MRI features of CSVD as per qualitative rating scales. Studies must include a rating system for presence and/or severity of the included imaging feature(s) and must report the prevalence/severity of the feature(s) separately for subjects with and without diabetes. Studies which do not report results separately for participants with versus without diabetes shall be excluded. Commonly used, validated rating systems (examples in Table 1) are preferred, but novel rating schemes will be deemed acceptable if they are adequately described as determined by consensus. Studies which use only a novel rating scheme that is not adequately described (by consensus) shall be excluded if attempts to contact the authors do not yield adequate clarification. If studies contain data regarding the presence and/or severity of cerebral atrophy, we will extract these data as well. However, studies reporting cortical atrophy alone are to be excluded because this finding in and of itself is not unique to CSVD.

### Types of studies

We will consider any studies that report MRI features of CSVD (at least one of white matter hyperintensities, lacunar infarcts, cerebral microbleeds, enlarged perivascular spaces) in adults with and without T2DM. To be eligible for inclusion, a study must report MRI findings for groups of participants both with and without diabetes mellitus. To maximize sensitivity, we will place no limitations on whether the imaging rater/s is/are blinded to clinical information, whether there is a measure of intra-rater/inter-rater reliability, or whether the level of experience or background (radiologist, neurologist, etc.) of the raters is specified though this information will be extracted if available and will contribute towards assessment of risk of bias. Studies will be observational; cohort, case control, and cross-sectional study designs will be eligible for inclusion. The anticipation is that the vast majority of these studies will be cross-sectional. We will only include studies reporting the presence versus absence of at least one of these four MRI changes in the total number of participants with versus without diabetes (2 × 2 table). These data are sufficient to calculate odds ratios [16].

## Methods

Adherence to preferred reporting standards for systematic review is confirmed with the PRISMA-P checklist (Additional file 1).

The study is registered with PROSPERO (CRD42016046669).

### Search method for identification of studies

Scoping searches were undertaken to ensure it was appropriate to commence a systematic review. A concise MEDLINE (Ovid) search resulting in 147 references which were reviewed by TS and CF. Records from the 17 papers they selected and five known papers were examined in MEDLINE (Ovid). The MeSH terms were reviewed and categorized which contributed to the conceptualization of the search and the construction of the draft search.

**Fig. 1:**
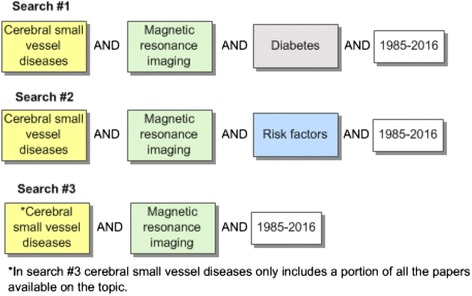
Schematic of search concepts

The intention of the review is to retrieve studies that include a population of adults with and without diabetes who have had a brain MRI examining the presence of CSVD. The most sensitive search would include the search concepts CSVD and MRI, but this results in an unmanageable number of results. Therefore, these two concepts will be combined with diabetes mellitus which should capture the majority of relevant papers. The above strategy may miss articles in which the study population is not described in the abstract of some papers or not all populations may be mentioned, therefore these papers, which could be missed using the will be captured through two other searches. Search two will include CSVD, MRI, and adverse effects. Search three will include CSVD but only studies where this is the main focus of the paper which will be combined with MRI. The three searches will be combined using the Boolean operator OR and duplicates will be removed. Together, the three searches should create a sensitive search strategy to capture relevant studies for this review (see Fig. 1). The subject headings of studies meeting the inclusion criteria will be examined to ensure all relevant terms have been captured. If needed, additional searches will be undertaken.

The initial search will be developed in MEDLINE and adapted for the following databases: Embase (Ovid), CINAHL (EBSCOhost) and Web of Science (Thomson Reuters). Results will be restricted to after 1985 as literature before 1985 would be prior to the clinical use of MRI. A language restriction shall not be applied to the search. If there are relevant non-English abstracts, attempts shall be made to translate them wherever possible. An illustration describing the search conceptualization and a draft search are included as Fig. 1 and Additional file 2.

In regard to grey literature, the proceedings of the International Stroke Conference, European Stroke Conference, European Stroke Organization, World Stroke Organization, the American Diabetes Association Scientific Sessions, the World Diabetes Congress, and the Annual General Meeting of the American Society of Neuroradiology will be searched. General searches through PapersFirst (WorldCat), ProceedingsFirst (WorldCat), and Web of Science (Thomson Reuters) will also be undertaken. The websites of pertinent organizations will also be examined for papers and the names of researchers.

Several approaches will be undertaken to increase our retrieval of relevant articles. The journals Stroke, International Journal of Stroke, Journal of Stroke and Cerebrovascular Diseases, Lancet Neurology, Diabetes, Neuroradiology, and American Journal of Neuroradiology will be hand-searched to ensure studies have not been missed. These journals are considered to be of the highest impact for the clinical subject of interest. Subject experts on CSVD will also be contacted to enquire about any studies felt to be applicable but not retrieved by our search strategy. Papers meeting the inclusion criteria will be searched in the Web of Science (Thomson Reuters) and Elsevier ScienceDirect for articles citing these papers. References from included papers will also be reviewed.

### Data collection

A record will be kept of all searches and search decisions to ensure reproducibility. Search results will be exported to a citation management program (EndNote ver. 7.0). Duplicates will be removed and retained separately. The resulting references will be exported separately to the two reviewers for independent review using MS Excel.

### Selection of studies

Two authors (CF, TF) will independently screen all titles and abstracts identified through the literature searches and will exclude all records clearly not meeting inclusion criteria. Disagreements will be resolved by consensus. The selection process will be pilot tested to ensure a high degree of agreement between reviewers. Full text of the remaining studies will then be retrieved. The same two authors (CF, TF) will independently assess the papers for fulfillment of inclusion criteria. In case of differences of opinion regarding study inclusion, a third review author (SY) will serve as arbiter. To avoid double counting, if multiple publications based on the same cohort of participants are retrieved, only the study reporting the largest sample size will be used. The reasons for excluding papers for which the full text was retrieved will be documented.

### Data extraction and management

A data extraction form will be used to collect details from the included studies. The form includes information on study design, patient population, and presence of neuroimaging features of interest (see Additional file 3). Two review authors (CF and TF) will independently extract the data. The data extraction form will be pilot tested on several papers to ensure consistency and that all relevant information is being captured. If necessary, a statistician will review the extraction of data to further ensure quality and reliability. Authors will be contacted for missing data.

We will extract the MRI features of interest (white matter hyperintensities, lacunar infarcts, cerebral microbleeds, and/or enlarged perivascular spaces, cerebral atrophy) accounted for in the study as well as any rating scales used. “Presence” versus “absence” of each feature will be determined as per Table 1. For studies using an alternate rating scale not included in Table 1, criteria for “presence” versus “absence” will be discussed between the raters (CF, TF) and with an additional expert if deemed necessary. For each feature included, we will extract 2 × 2 tables (presence versus absence of MRI findings in subjects with versus without diabetes) from publications, or we will reconstruct these from data in the publication. For studies where the data does not permit a 2 × 2 table construction, we will contact the study authors to ask for the relevant data before deciding whether or not to exclude the study from the systematic review. Presence of adjustment for relevant covariates (i.e., smoking status, age, hypertension, dyslipidemia) and the covariates themselves included in the analysis will be recorded.

### Assessment of methodological quality

We will assess the methodological quality of each study using the Quality Assessment Tool for Observational Cohort and Cross-sectional Studies from the National Heart, Lung and Blood Institute [17]. This tool was designed to provide a framework to focus on the key concepts for establishing the internal validity of cohort and cross-sectional studies. Use of a more rigorous assessment tool is precluded by the primarily cross-sectional nature of our data.

Two authors (CF, TF) will assess methodological quality independently and will resolve all disagreements through discussion or with arbitration by a third author (SY).

### Data synthesis and statistical analysis

We anticipate that there may be significant heterogeneity in the prevalence of MRI features of CSVD in subjects with versus without diabetes across studies. There are several factors that could contribute to such heterogeneity. These factors include the following: differences in demographic and clinical features (e.g., age, hypertension, renal disease, smoking, duration and severity of diabetes) among study cohorts; differences in definitions of diabetes; technical differences between MRI scanners and acquisition protocols (e.g., magnet strength, slice thickness, sequences); use of different radiological rating scales; and differences in MRI rater skill and reliability. An I^2^ statistic will be calculated for the studies to be included in each proposed meta-analysis (i.e. for each neuroradiologic correlate of interest) with values of 25, 50, and 75% suggesting low, moderate, or high degrees of heterogeneity, respectively [18].

Scales which report a dichotomized (i.e., present or absent) or categorical (i.e., absent, mild, moderate, severe) shall be harmonized for meta-analysis if deemed appropriate by our statistician. Other types of rating scales shall not be included in a meta-analysis and the data based on any such data scale would be presented in narrative form.

If significant heterogeneity between studies, as determined by consultation with our statistician, prevents meaningful pooling of the data, we will limit ourselves to providing a narrative description of observed trends. This would include a summary of the design of each of the studies reporting the prevalence of each CSVD feature and the odds ratio for presence of that feature from each individual study.

If some studies (at least two) may be pooled into a meta-analysis, then for each CSVD feature we will calculate odds ratios and 95% confidence intervals for the presence of the features in subjects with versus without diabetes from the 2 × 2 tables extracted from the data collection forms. Given the heterogeneity of the populations studied, assumption of a fixed effect size across populations would not be justified, thus analyses would be performed using a random effects model [19]. Given the dichotomized (presence or absence) or categorical (severity measure) nature of our data of, meta-analysis will be performed using the Mantel-Haenzel method [20].

If there are sufficient data to allow such analyses (in principle from as few as a single high quality study, but if possible by pooling data from multiple studies), we will perform subgroup analyses for participants with renal disease and participants with hypertension. In addition, if sufficient data are available, we shall perform subgroup analyses by age and diabetes duration.

For diagnostic studies, knowledge of the mechanisms that may induce publication bias or empirical absence for its existence is sparse (in contrast to intervention studies), however, we will attempt to assess for publication bias using Deeks’ test [21]. Funding sources and conflict of interest will be extracted from included studies.

Statistical analysis will be performed using RevMan software [22].

### Quality of evidence

A summary of findings table will be created. We will use the Grading of Recommendations, Assessment, Development and Evaluation (GRADE) approach to assess the quality of evidence for the primary outcomes of interest [23].

## Discussion

The neuroradiologic correlates of CSVD have only been fully described in the last decade. Much research still remains into how the risk factors for CSVD lead to the emergence of the particular pathologic and neuroradiologic correlates. Patients with diabetes mellitus represent a large portion of adults with CSVD. Although any effects of elevated glucose may be “dose-dependent” (i.e., dependent on severity and duration of diabetes), we hypothesize that even dichotomized categorization of participants into those with versus without diabetes will reveal significant differences between these two groups, when adjusted for confounding risk factors such as age or history of hypertension.

Whether or not such a distinct phenotype is uncovered by this review, additional analyses examining other CSVD risk factors (i.e., hypertension) and their relationship to a distinct phenotype would further clarify possible mechanisms of CSVD and help to guide targeted therapeutic strategies.

## Additional files

Additional file 1: PRISMA-P checklist. (DOCX 32 kb)
Additional file 2: Search strategy. (DOCX 14 kb)
Additional file 3: Data collection form. (DOCX 49 kb)

## Abbreviations

CSVD: Cerebral small vessel disease
EMBASE: Excerpta Medica database
FLAIR: Fluid-attenuated inversion recovery
MeSH: Medical subject heading
MRI: Magnetic resonance imaging
SWI: Susceptibility-weighted imaging
T2DM: Type 2 diabetes mellitus
