# Supplementary material for: What is the relationship between type 2 diabetes mellitus status and the neuroradiological correlates of cerebral small vessel disease in adults? Protocol for a systematic review
Source: Syst Rev. 2017 Jan 17;6:7. doi: 10.1186/s13643-017-0410-1 (PMC5240395; doi:10.1186/s13643-017-0410-1)
Supplement: Additional file 2: — Search strategy. (DOCX 14 kb) [file 13643_2017_410_MOESM2_ESM.docx]

## Addition File S2

Database: Ovid MEDLINE(R) In-Process & Other Non-Indexed Citations and Ovid MEDLINE(R) <1946 to Present>

Search Strategy:

-----------------------------------------------------------

1 Cerebrovascular Disorders/ (44351)

2 limit 1 to yr="1995 - 2011" (13388)

3 exp basal ganglia cerebrovascular disease/ (427)

4 exp brain ischemia/ (89006)

5 exp carotid artery diseases/ (40922)

6 exp cerebrovascular trauma/ (5977)

7 exp dementia, vascular/ (5743)

8 exp intracranial arterial diseases/ (52156)

9 exp intracranial arteriovenous malformations/ (7604)

10 exp "intracranial embolism and thrombosis"/ (19408)

11 exp intracranial hemorrhages/ (60196)

12 leukomalacia, periventricular/ (1332)

13 sneddon syndrome/ (165)

14 exp stroke/ (99743)

15 susac syndrome/ (107)

16 vascular headaches/ (1300)

17 exp vasculitis, central nervous system/ (7110)

18 vasospasm, intracranial/ (2609)

19 cerebral small vessel diseases/ (272)

20 cadasil/ (601)

21 cerebral amyloid angiopathy, familial/ (56)

22 fabry disease/ (2838)

23 melas syndrome/ (1079)

24 microscopic polyangiitis/ (342)

25 stroke, lacunar/ (295)

26 cerebral small vessel disease?.tw. (640)

27 Leukoaraiosis/ (388)

28 Leukoaraiosis.tw. (810)

29 lacunar infarct$.mp. (2112)

30 enlarged perivascular space?.mp. (62)

31 hyperintensit$.mp. (5904)

32 hyper intensit$.mp. (143)

33 WMH$.mp. (1165)

34 (T2 adj35 (MR or MRI or Magnetic resonance)).mp. (26788)

35 microbleed$.mp. (1183)

36 (lacun$ adj8 (infarct$ or stroke or hyper$ or white matter)).mp. (3649)

37 Brain/ (418651)

38 White Matter/ (1818)

39 cerebral arteries/ (15284)

40 frontal lobe/ (27126)

41 basal ganglia/ (11941)

42 hippocampus/ (87774)

43 brain diseases/ (50969)

44 white matter.mp. (42049)

45 *Cerebrovascular Disorders/ (30791)

46 *cognition disorders/ (39527)

47 or/37-46 (654456)

48 pa.fs. [pathology] (2420591)

49 47 and 48 (148922)

50 perivascular space?.mp. (1508)

51 ((Virchow or Virchow's) adj5 space?).mp. (431)

52 vascular lesion?.mp. (9251)

53 or/2-36,49-52 [CSVD](438330)

54 magnetic resonance imaging/ or cholangiopancreatography, magnetic resonance/ or diffusion magnetic resonance imaging/ or echo-planar imaging/ or fluorine-19 magnetic resonance imaging/ or magnetic resonance angiography/ or magnetic resonance imaging, cine/ (344637)

55 MRI.mp. (169203)

56 magnetic resonance imag$.tw. (164680)

57 diffusion tensor imaging/ or functional neuroimaging/ or brain mapping/ or connectome/ (79996)

58 Magnetic resonance imaging.kw. (6800)

59 or/54-58 [MRI](481395)

60 53 and 59 [CSVD & MRI](105281)

61 diabetes mellitus/ or diabetes mellitus, type 1/ or wolfram syndrome/ or diabetes mellitus, type 2/ or diabetes mellitus, lipoatrophic/ or diabetes, gestational/ or diabetic ketoacidosis/ or donohue syndrome/ or prediabetic state/ (255746)

62 Diabetes Complications/ (37519)

63 diabetes.kf. (36729)

64 (T2DM or T2D).mp. (13819)

65 diabetes.tw. (378853)

66 or/61-65 [Diabetes](461308)

67 53 and 59 and 66 [CSVD & MRI & DM] (1794)

68 *risk factors/ or *Disease Susceptibility/ or *Age Factors/ or *Aging/pa or *time factors/ (15922)

69 53 and 59 and 68 [CSVD & MRI & Risk] (877)

70 exp *Cerebral Small Vessel Diseases/ (4558)

71 59 and 70 [MRI & CSVD] (956)

72 67 or 69 or 71 [All 3 searches](3550)

73 animals/ not (animals/ and humans/) (4191698)

74 72 not 73 (3461)

75 comment/ or editorial/ or letter/ or news/ (1633427)

76 74 not 75 (3347)

77 limit 76 to yr="1985 -Current" (3346)

78 limit 77 to English language (3013)

79 77 not 78 [non-English] (333)
