# Supplementary material for: What is the relationship between type 2 diabetes mellitus status and the neuroradiological correlates of cerebral small vessel disease in adults? Protocol for a systematic review
Source: Syst Rev. 2017 Jan 17;6:7. doi: 10.1186/s13643-017-0410-1 (PMC5240395; doi:10.1186/s13643-017-0410-1)
Supplement: Additional file 3: — Data collection form. (DOCX 49 kb) [file 13643_2017_410_MOESM3_ESM.docx]

Rater initials

PMID

Year

Population:

Country

Setting

Definition of Diabetes:

Criteria

Assessment of duration of disease?

Assessment of severity of disease and how?

Timing:

MRI

Scanner type

Sequences and acquisition protocols

Salient co-variates compared between diabetics/non-diabetics?

Age

HTN

Dyslipidemia

Smoking

Renal failure

Other

Analysis accounts for covariates?

Neuroimaging feature(s) in study (print one copy of page 2 for each separate neuroimaging feature)

WMH

Lacune

CMB

PVS

cerebral atrophy

**Neuroimaging feature of CSVD:**

WMH/ lacune/ CMB / PVS / cerebral atrophy (circle)

Rating scale used

Rater characteristics:

Number of raters

Training (neuroradiologist, neurologist, trainee, etc)

Measure of intra/inter-rater reliability and how?

Presence/absence of neuroimaging feature

|  | DM- | DM+ | Total |
| --- | --- | --- | --- |
| MRI feature present |  |  |  |
| MRI feature absent |  |  |  |
| Total |  |  |  |

Severity of feature – measure 1 (specify)

|  | DM- | DM+ | Total |
| --- | --- | --- | --- |
|  |  |  |  |
|  |  |  |  |
| Total |  |  |  |

Severity of feature – measure 2 (specify)

|  | DM- | DM+ | Total |
| --- | --- | --- | --- |
|  |  |  |  |
|  |  |  |  |
| Total |  |  |  |

Severity of feature – measure 3 (specify)

|  | DM- | DM+ | Total |
| --- | --- | --- | --- |
|  |  |  |  |
|  |  |  |  |
| Total |  |  |  |
